# Texas State Medical Association—Thirty-Second Annual Meeting

**Published:** 1900-05

**Authors:** 


### The Medical Societies.

### Texas State Medical Association—Thirty-Second Annual Meeting.

### FIRST DAY.—*Morning Session.*

AUDITORIUM, WACO, TEXAS, April 24, 1900.

The Association was called to order at 11 a. m., by Dr. J. H. Sears, chairman of the committee of arrangements.

Rev. Dr. Frank Page, Rector of St. Paul's, delivered the invocation.

An address of welcome was delivered by Hon. J. W. Riggins, Mayor of Waco.

Dr. Sears then introduced Judge John G. Winter, of Waco, who delivered, on behalf of the citizens of Waco, an address of welcome.

Dr. J. H. Sears then addressed the Association on behalf of the Central Texas Medical Association.

The President, Dr. A. B. Gardner, responded to the addresses of welcome, as follows:

"*Mr. Chairman, Ladies and Gentlemen:*

"As the representative of this Association I thank the speakers for the kind welcome that we have received from the citizens of Waco. We of this Association who have attended meetings here on previous occasions well know the genuineness of the welcome that we always receive in the good city of Waco. It is not an empty welcome of words that comes to us here, but we are made to feel at home, and that we are among our friends, and that they are glad to have us among them. It has been my pleasure to have been here on two previous occasions at meetings of this kind. I know from experience that some remarks of the mayor are very applicable. He wishes us to put the police to sleep, but I think the doctors will hardly be in a condition, after drinking this artesian water (if that is what they drink), to put anybody to sleep, and some of them may want to go to sleep on the streets or anywhere else. I feel that when we come to Waco we are going to have a good time, and I feel that upon this occasion more especially we will have a pleasant and profitable meeting, and that all will be done to make this a pleasant visit that can be done by the citizens of Waco. In behalf of the Association I again thank you for the welcomes extended to us."

The roll was called and a quorum was found to be present.

The President took the chair and the meeting was declared open for business.

Upon motion, the reading of the minutes was dispensed with.

Dr. J. C. J. King, associate chairman of the committee of arrangements, announced the program for the social features of the meeting, receptions, etc.

The annual report of the Secretary was read, as follows:

### Secretary's Annual Report.

*Mr. President and Gentlemen:*

I beg to present herewith my annual report and incorporate with the same a statement concerning publication of the Transactions for 1899.

#### MEMBERSHIP.

On the roll, Transactions of 1899: Honorary, 27; ordinary, 266; subsequently added, 6; total, 299. Died, 2. Total now enrolled, 297.

Elected at San Antonio, April 25-29, 1899: Ordinary, 43.

Dropped from roll since 1898: By death, 7; from non-payment of dues, 93; total dropped, 100.

#### DEATHS SINCE LAST REPORT.

Dr. M. D. Knox, Hillsboro, died August 27, 1899.

Dr. J. M. Litten, Austin, an honorary member, died March 31, 1900.

If any other deaths have occurred they have not been brought to my notice.

#### THE TRANSACTIONS FOR 1899.

The edition of 1898 of 390 copies proving to be inadequate to supply the demand, I decided to issue 500 copies of the proceedings of 1899, Von Boeckmann, Schutze & Co., of Austin, agreeing to furnish that number at the same price they had charged for the 390 copies, it was not thought necessary to ask for additional bids, experience having demonstrated their ability to bid lower than anyone else. The contract was awarded to them as follows:

| | |
|---|---|
| 490 copies, cloth bound: Brevier | $1.03 per page. |
| Long primer | .96 per page. |
| Morocco bound, 10 copies | .60 per volume. |

The publishers' bill is as follows:

To 500 copies Transactions:

| | |
|---|---|
| Long primer, 234 pages | $224 64 |
| Brevier, 128 pages | 131 84 |
| Tabular matter | 7 21 |
| 10 copies, Morocco bound | 6 00 |
| Wrapping, mailing, addressing | 10 00 |
| Cuts | 3 50 |
| | $383 19 |
| Postage | 58 80 |
| | $441 99 |

Cost per volume, about 70 cents.

Cost per volume, delivered, about 88 cents.

The books were ready for distribution the latter part of October, and payment promptly made by the Treasurer. There are now on hand 74 copies, showing distribution of 426. I have had numerous requests from public medical libraries throughout the country for files of our transactions, with which I have complied so far as I was able, charging only the actual cost of boxing. The volume of last year was somewhat diminished in size by the failure of several authors to furnish me with their papers. There is no excuse for this flagrant violation of an imperative rule of the Association, which requires that all papers whose titles appear upon the programme shall be handed to the Secretary, as authors are permitted to publish them elsewhere prior to the publication in the Transactions, if they so desire.

My account with the Association since last report is as follows:

H. A. West, in account with the Texas State Medical Association:

| | | | | |
|---|---|---|---|---|
| 1899. | DR. | | | |
| June 10. | W. L. Rogers, dues | $5 00 | | |
| Dec. 29. | T. J. Tyner, dues | 7 00 | | $12 00 |
| | CR. | | | |
| June 22. | Postage | $1 00 | | |
| July 25. | Expen. on copy | 50 | | |
| July 15. | Postage | 75 | | |
| Aug. 5. | Knapp Bros., stationery | 3 00 | | |
| Oct. 26. | Freight and drayage on Transactions | 2 50 | | |
| Dec. 1-April 10. | Postage | 2 75 | $10 50 | |
| 1900. | | | | |
| | Balance due the Association | | 1 50 | $12 00 |

FORFEITURE OF CHARTER.

In order that there may be no misunderstanding of the matter, I will mention in detail the circumstances whereby the charter of the Association was forfeited. Sometime during the latter part of May I received a note from the Secretary of State informing me that in consequence of failing to pay the franchise tax of $10.00 by May 1, unless a heavy penalty was paid, that our charter would be taken from us. This communication, which had been directed to Austin, was the first I had received. I wrote to Mr. Hardy, explaining that it had been the custom to send me notice prior to the annual meeting the last week in April, and that as no such notice had been received the matter had escaped my attention, and requested that the penalty be remitted. Upon his declining to do so, I consulted the President, Dr. Gardner, and upon his advice sent a check for $10.15, May 29, 1898. On June 9 the check was returned with the information that the charter of the Association was forfeited, and that it was denied the rights of a corporation in Texas. I have never seen the advantage of paying the annual franchise tax of $10.00 to the State, and will leave the matter to such adjudication as your wisdom may dictate.

AFFILIATING SOCIETIES.

It will be remembered that at the last meeting of the Association resolutions were adopted establishing the relation of local societies with this body upon a basis of $5.00 annual dues for each twenty members, or fraction thereof, entitling them to representation in the same proportion. Due notice was given of this action, but, so far as I have been informed, only one society, viz.: the South Texas, has paid its dues, and is entitled to send delegates.

Presiding at the last meeting of that body, I appointed the following delegates, who are entitled to admission at this meeting without payment of dues:

Drs. H. A. Barr, Beaumont; J. T. Moore, Galveston; O. S. Hodges, Galveston; J. E. Cannon, Alvin; G. D. Martin, Beaumont.

Some definite settlement of this vexed question should be made at this meeting.

THE FINANCIAL STATUS.

I am informed by the Treasurer that he has on hand about $1100.00. This splendid showing is the result of the adoption of business methods in the collection of dues; so in place of passing around the hat to receive money to pay our debts we will have to take into consideration the investment of our surplus funds.

Respectfully submitted,
H. A. WEST, Secretary.

Upon motion, the report was referred to the following committee: J. T. Wilson, J. M. Fort, H. P. Cooke.

The Treasurer read his annual report as follows:

TREASURER'S REPORT.

R. F. Miller, Treasurer, in account with the Texas State Medical Association:

RECEIPTS.

| | | |
|---|---|---|
| April 24, 1898. | To balance on hand as per annual report....... | $ 414 56 |
| April 28, 1899. | To dues collected during San Antonio meeting.. | 990 17 |
| April 21, 1900. | To dues collected since last meeting........... | 708 65 |
| | | $2113 38 |

DISBURSEMENTS.

| | | |
|---|---|---|
| 1899. | | |
| April 27. | By cash paid H. A. West, Secretary's salary........ | $ 300 00 |
| April 27. | By cash paid J. Larendon, Treasurer's salary....... | 75 00 |
| April 27. | By cash paid C. W. Howth, stenographer............. | 35 00 |
| April 27. | By cash paid U. S. postage......................... | 10 00 |
| April 27. | By cash paid W. W. Cunningham, Memorial Board of Health.................................... | 5 00 |
| April 27. | By cash paid porter at Turner Hall................. | 1 00 |
| April 28. | By cash paid stenographer for lists................ | 50 |
| May 15. | By cash paid U. S. postage and stationery.......... | 1 59 |

| | | | |
|---|---|---|---|
| June | 1. | By cash paid Von Boeckmann, Schutze & Co., memorial and address | 15 00 |
| June | 1. | By cash paid paper, envelopes and printing for Treasurer | 9 00 |
| June | 7. | By cash paid U. S. postage on 250 notifications | 5 00 |
| June | 13. | By cash paid U. S. postage | 3 00 |
| June | 15. | By cash paid C. W. Howth, stenographer, balance | 24 25 |
| July | 1. | By cash paid exchange | 40 |
| July | 5. | By cash paid U. S. postage | 2 00 |
| July | 6. | By cash paid revenue stamps on 176 drafts, etc | 4 00 |
| Aug. | 5. | By cash paid U. S. postage | 5 00 |
| Sept. | 10. | By cash paid U. S. postage | 5 00 |
| Sept. | 11. | By cash paid fees H. B. Powell (dec'd) returned | 7 50 |
| Oct. | 5. | By cash paid Von Boeckmann, Schutze & Co., postage Transactions | 58 80 |
| Oct. | 23. | By cash paid Von Boeckmann, Schutze & Co., Transactions | 383 19 |
| 1900. | | | |
| Feb. | 1. | By cash paid U. S. postage | 5 00 |
| April | 24. | By cash balance on hand | 1159 24 |
| | | | $2113 38 |

Respectfully submitted,

R. F. MILLER, Treasurer.

DR. WEST: I would like for the Treasurer to specify the amount received by him from the South Texas Medical Association.

DR. MILLER: Twenty-five dollars.

DR. WEST: I would like to ask if any other association paid its dues?

DR. MILLER: No, sir.

Upon motion, the Treasurer's report was referred to the same committee to which the Secretary's report had been referred.

### PRESIDENT'S ANNUAL MESSAGE.

The President then read his annual message and recommendations, as follows:

"*Gentlemen of the Texas State Medical Association:*

"I would be less than human if I should not be filled with emotions of pride upon greeting you on this auspicious occasion. There has never been a meeting of this Association under more favorable circumstances than this, our thirty-second. And it is the prayer of your humble servant that you may have a most profitable, harmonious and pleasant meeting in this beautiful and hospitable city, and that all of your deliberations may tend to strengthen the bonds of unity and good will which at present pervade our Association.

"For the past two years our financial condition has been steadily improving, and is being conducted on strict business methods. Instead of having to ask contributions from our members to defray the expenses of the Association, the reports of our Secretary and Treasurer show a very handsome cash balance on hand after having paid all expenses for the past year, including the publication of our Transactions, for which I must compliment the publishing committee on the very neat and complete volume they have furnished the members of the Association.

"During the past year there has been only an extra session of the Legislature, during which no medical legislation could be expected, and your legislative committee made no effort to obtain any. I would recommend that this committee be continued. Also I would respectfully urge upon every member of this Association and every regular physician in the State the necessity of their co-operation with this committee by calling the attention of their respective candidates for State Senator and Representative to the need of some medical legislation, and urge them to support the bill for the regulation of the practice of medicine, which will be presented for the consideration of the Legislature by said committee, and which has the endorsement of this Association; also the necessity of a State Board of Health and Vital Statistics. With the united efforts of the physicians much can be accomplished for the good of the State and the elevation of the standard of medicine.

"Another very important subject is the organization of county and district medical societies so happily begun, and I hope will be continued and fostered in every manner possible by this Association. In this connection allow me to call your attention to a resolution which was adopted on the last day of our meeting in 1899, requiring auxiliary societies to pay five dollars annual dues to this Association for every twenty members or fraction thereof.

"My observation leads me to believe this to be unwise, and I would suggest the reconsideration of the resolution, as it may tend to defeat the object sought, the unification of all societies in the State Association.

"Since our last meeting in San Antonio there has been chartered by the State and located at San Antonio the 'New York Medical College,' which, from its advertisements and announcements, has every appearance of a bogus concern or diploma mill. I would suggest that this Association adopt some method to investigate this institution, and should it prove a fraud every honorable means be used to drive it from the State. I feel certain that it is not the intent of our laws to charter institutions to perpetrate fraud on its citizens, or assist others in doing so.

"Last June our Secretary called my attention to the fact that the Secretary of State had failed to notify him when our franchise tax was due, and when the tax was tendered the State demanded a heavy fine, as the charter was forfeited. I suggested he should await this meeting and submit the matter to the Association whether there was any benefit to be derived from having a charter.

"Gentlemen, you are to be congratulated upon the very comprehensive program which has been prepared for you. There are many important subjects presented, and by able gentlemen, and much time has been devoted to scientific work. In this way we may expect the Association to attract many more of the able men of the profession in the State to it, and in this way build up the best State association in the Union.

"In conclusion, and with profound sorrow, I refer to those of our members who, since our last meeting, have answered the Master's call, and have gone from among us to the shores of the great unknown.

"Dr. M. D. Knox died August 25th, 1899, at his home in Hillsboro. His familiar face and form were ever with us at our meetings. No one loved this Association more than he; faithful to every duty assigned him; a pure Christian gentleman and accomplished and conscientious physician; a true and devoted friend ready to sacrifice his own ambitions to those he claimed as friends.

"Dr. J. M. Litten, an honorary member, died March 31st, 1900, at a ripe old age, at his home in Austin. He was one of the Nestors of medicine in the State, and had seen much of the vicissitudes of the pioneer doctor's life in Texas. Those who knew him best loved him most.

"I beg you, gentlemen, to accept my thanks for your indulgence and kind consideration of me, and assure you of my high appreciation of your confidence and esteem."

Upon motion the President's address and recommendations were referred to the following committee: S. C. Red, Sam R. Burroughs, and Taylor Hudson.

F. M. PITTS, of Mt. Calm: The President of our State University, Col. W. L. Prather, is with us, and I move that all the honors and privileges of this Association be tendered to him, and that he be introduced to the Association.

The motion was unanimously carried.

Col. W. L. Prather, President of the University of Texas, was introduced to the Association by the President, Dr. Gardner, and spoke as follows:

ADDRESS OF COL. W. L. PRATHER.

"*Mr. President and Gentlemen of the Association:*

"I find myself in rather a difficult position, and I do not wonder at the President's difficulty in establishing whether I be a doctor or a Texas colonel. I have already felt that it was a compliment in traveling among strangers, that I have frequently been taken for a doctor. I have never known the basis of that supposition, but still I have always considered it as complimentary. I am glad to be with the representatives of this great Association—great not only because of the men who constitute its membership, but great on account of the great responsibilities that rest upon them as the representatives of the health of the people of this great commonwealth. I am glad to appear before them as being specially allied to their vocation by reason of my acceptance of the presidency of the University of Texas, including the medical college as a branch thereof, at Galveston, Texas. I was glad to hear the allusion of your president to the proper investigation and arraignment of that fraud which is located at San Antonio. I was glad to read in the morning paper that the Attorney-General of the State of Texas was taking active measures to suppress it. I have felt that not only the medical profession but the legal profession were entitled to that legislation which would prevent the people from suffering from ignorance, and ignorance, too, in lines where investigation could not be made. It has been said when I have gone before the committees of the Legislature, representing the Texas State Bar Association, and we met men who had no special training in the law upon the committees to which these bills were referred, that the lawyers could take care of themselves, and they might take care of themselves, and generally the result was to pigeon-hole those bills. That is true in a certain sense, but in a very limited sense. It is not true at all of the medical profession. I remember the great doctor, Richard McCulloch, who was formerly a professor of physics in the university at Washington, was being examined in a celebrated murder case at Baltimore. He was called doctor, but insisted that he was simply a chemist, and not a doctor of medicine. When he was examined by Tekle S. Wallace, and was asked the question and alluded to as 'Doctor' by the lawyer that sought to get the better of him, he was asked: 'Doctor, the mistakes of physicians are frequently buried under ground, ain't they?' He said, 'Yes; and those of lawyers are frequently hanged on trees.' The medical profession does need legislation in this State. It is an outrage upon the citizenship of Texas that the lives and health of our people shall be committed to ignoramuses. While the State of Texas is expending thousands of dollars, and doing it wisely, in the protection of the cattle of Texas, it ought to do something for the protection of the people of Texas. And it lies, gentlemen, in your power to accomplish that. if you will but organize and direct intelligently and with determination your force. The medical college at Galveston, and the general interests of the University of Texas, desire to join hands with you in an earnest effort to uplift and establish upon a permanent basis the profession of medicine, a basis where the people can trust it, where the citizenship of Texas can firmly rely upon the man who carries his degree as a doctor of medicine that he is in truth and in fact prepared for his vocation.

"I am glad to meet you in this, my home. I have gone from it with many, many regrets. I have lived here since I was a child. I came here in 1854, and there are very few of those that were engaged in the profession then now here upon this stage. We have one who has been your President, and who spoke so feelingly to the physicians, so earnestly, awhile ago. The one incident in his life that I have often recalled on other occasions is the night in the long ago when he took his own bride. That night he received a message that a man's life was hanging in the scales in the county of Limestone, and leaving her whom he loved more than all others he went that night, when we did not have railroads, and rode in the hours of midnight, and stayed a week with that man, and saved his life. When I heard his touching words today I could not help but wonder how many young physicians belonging to this Association would imitate that Roman spirit today.

"Texas owes a great deal to the noble character of its doctors. They have been in the discharge of public and private duty a great force for good and for the advancement of mankind. The last great president of the Republic of Texas was a distinguished doctor. I would not undertake on this occasion to recount the many glorious deeds and the rich heritage which you, as their successors, have in the grand examples which have gone before. I feel that Texas, as she has done in the past, must in the future uphold the standards for high manhood, for high professional conduct, and for the advancement of civilization. I am glad to look into your faces and meet you on this occasion."

Upon motion the meeting adjourned until 2:30 p. m.

### FIRST DAY.—*Afternoon Session.*

The Chair declared the Section on General Medicine open, H. P. Cooke, of Galveston, Chairman, and S. C. Red, of Houston, Secretary.

The Chairman of the Section presented his address on "Treatment of Typhoid Fever."

Dr. West moved that the rules providing that the chairman's address shall not be discussed be suspended, and that Dr. Cooke's paper be open for discussion.

The motion carried.

The Judicial Council reported favorably upon a list of names for membership, which, upon motion, was adopted.

Dr. S. C. Red, of Houston, read a paper entitled "Observation Upon a Recent Epidemic of Scarlet Fever in Houston."

On motion, the paper was received and opened for discussion.

T. M. Wilson, of Thornton, read a paper entitled "Croupous Pneumonia," which was, on motion, received and opened for discussion.

Dr. Malone Duggan, of Eagle Pass, read a paper entitled "Leprosy—Report of a Case," which was also received and opened for discussion.

Dr. J. D. Osborn, of Cleburne, read a paper entitled "The Treatment of Tuberculosis with Watery Extract of Tubercle Bacilli."

There being other papers on "Tuberculosis," it was moved and carried that the discussion be deferred until all the papers were presented.

The Secretary read the following paper by Dr. D. R. Fly, of Amarillo: "Tuberculosis—Its Prevention and Treatment."

Dr. J. A. Rawlings, of El Paso, read a paper entitled "Climatic Conditions in Western Texas, and their Influence Upon Pulmonary Conditions."

On motion, the discussion of these papers was postponed, and the meeting adjourned until 8 p. m.

### FIRST DAY.—*Night Session.*

At 8 p. m. the meeting reconvened and the Section on Obstetrics and Diseases of Children was declared upon, the chairman, J. J. Roberts, of Hillsboro, presiding.

The Chairman presented his report.

Dr. J. A. Rawlings, of El Paso, presented a paper entitled, "Anæsthesia in Labor," which was received and discussed.

Dr. H. A. West, of Galveston, read a paper entitled "Report of a Case of Cerebral Meningitis Following Measles—Recovery," which was, on motion, received and discussed.

Dr. Taylor Hudson presented a paper on "Diagnosis," which was received and discussed.

The convention then adjourned until 9 a. m. April 25th.

SECOND DAY.—*Morning Session.*

The President called the convention to order.

The report of the Committee on the Secretary's Report was read and adopted.

The report of the Committee on the Treasurer's Annual Report was read and adopted.

The report of the Committee on the President's Annual Report and Recommendations was presented as follows:

"WACO, TEXAS, April 24, 1900.

"*Mr. President:*

"Your Committee on the President's Message and Recommendations beg leave to make the following report:

"First. That the Association authorize the President to continue the Committee on Medical Legislation, and that the members pledge themselves to give all possible support to the carrying out of the objects of the said Legislative Committee.

"Further, that a majority of your committee recommend the abolishing of the resolution adopted at San Antonio regarding the affiliating medical societies, and in lieu of said resolution substitutes the following: That county and district associations of this State may come into affiliation with this Association by paying..........initiation. Said society shall be entitled to send one delegate for every twenty members or fraction thereof.

"We approve of the recommendations of the President, and commend the action taken by the Attorney-General of Texas in the case of the New York Medical College of San Antonio, Texas.

"Your committee would beg to say that in their opinion it is to the interest of this Association that our Secretary be authorized to re-charter, or reinstate, the charter of this Association.

"And further, that we concur in the expression of our President in regard to the death of our deceased members, Dr. M. D. Knox and Dr. J. M. Litten.

"Respectfully submitted,

"SAM R. BURROUGHS,

"S. C. RED,

"TAYLOR HUDSON."

Dr. West moved that the foregoing report be adopted, except the clause which relates to affiliating societies, and that the consideration of that clause be deferred until the report of the Committee on Medical Societies is presented, when the entire matter as contained in both reports may be considered together.

A substitute was offered that the report of the Committee on President's Message be laid upon the table until the report of the Committee on Medical Societies is heard.

The substitute prevailed.

The report of the Committee on Medical Societies was presented, the reading of the reports of societies being, on motion, dispensed with.

It was moved that the report be adopted and that a vote of thanks be tendered the committee for its efficient work.

The motion prevailed.

[All discussions omitted for lack of space.—ED.]

Dr. Wilson moved that the Committee on County and District Society Organizations be continued.

Dr. West moved that the report of the Committee on President's Report and Recommendations be taken from the table and brought before the house.

The motion prevailed.

Dr. Osborn moved the adoption of the report with the insertion of $2.50 from each society as an initiation fee.

[The result of the discussion was that all tax was removed from affiliated societies.—ED.]

DR. OSBORNE: Dr. Wilson is chairman of the Committee on Medical Legislation, and wishes to read his report now, as he has to leave. I move that the rules be suspended and this report be read.

The motion prevailed.

[Dr. Wilson's report will be published in a subsequent number of the JOURNAL.—ED.]

### SECOND DAY.—*Afternoon Session.*

Dr. West moved that Dr. A. Nolte, of the Louisiana State Board of Health, be extended the privileges of the floor.

Motion carried.

Dr. T. W. Shearer, Chairman of the Section on State Medicine and Public Hygiene, being absent, Dr. Red called the Section to order and acted as chairman.

Dr. H. A. West read a paper entitled "Maladministration of Public Medical Affairs in the State of Texas." [Published herewith.]

On motion the paper was received and opened for discussion.

[The discussion on Dr. West's paper and on the subject of State Medicine generally is omitted for want of room, and will be published later in pamphlet form and in the Transactions.—Ed.]

The Section on Surgery was then declared open. It was announced that Dr. B. F. Kingsley, chairman of the Section, would not be present until night, having been detained by a wreck.

It was moved that Part II of the Section on Surgery be taken up, and that Dr. J. M. Fort preside. The motion prevailed.

Dr. Bacon Saunders, of Fort Worth, read a paper entitled "Treatment of Fracture in the Neighborhood of the Shoulder, Elbow and Wrist Joints."

On motion, the paper was received and opened for discussion.

A report from the Judicial Council was received on motion.

Dr. J. E. Thompson presented a paper entitled "Three Cases of Closure of Jaws, Produced by Mercurial Stomatitis." [Published herewith.—Ed.]

On motion, the paper was received and opened for discussion.

On motion the meeting adjourned until 2:30 p. m.

### Second Day.—After Discussion.

The motion to endorse the Atlanta regulations was then carried.

It was moved and carried that a committee be appointed, consisting of Drs. Harrison, Daniel and Massey, to utilize the paper of Dr. West, the address of Dr. Smith, and the discussion on same as they think advisable, and that it be left to the discretion of the committee as to any expenditure in connection with it.

It was moved that a committee of three be appointed to draft resolutions to be forwarded to Texas representatives in Congress, protesting against the passage of the anti-vivisection bill.

The motion prevailed.

The Chair appointed Drs. Wooten, West, and M. M. Smith on said committee.

A report of the Judicial Council was received.

It was moved and carried that the rules be suspended and there be no night session tonight, and that all Sections be moved up one number.

The convention then adjourned to meet April 26, 1900, at 9 a. m.

### THIRD DAY.—*Morning Session.*

It was moved and carried that Dr. H. L. Tate, of Lindale, be placed on the honorary roll of the Association.

Dr. Bell offered the following resolution:

"*Resolved,* That this Association appropriate as much as $50.00 to each congressional district, to be used at the discretion of the Committee on Organization, as they see proper, and if necessary."

The Judicial Council reported, recommending Dr. Barr, of Beaumont, for membership. The report was received and adopted.

It was moved and seconded that $200.00, or as much thereof as may be necessary, be appropriated for the expenses of the campaign committee.

The motion prevailed.

DR. PASCHAL: I have been delegated by the West Texas Medical Association to present to this body the necessity of a home, State or national, for indigent consumptives, and if the Chair will allow me I will read the report which I was requested to make to this Association.

[Report will be published later.—ED.]

Dr. Paschal then presented the following resolution:

"WHEREAS, Tuberculosis is one of the most fatal of the contagious diseases, and is the cause of about one-sixth of the deaths from all causes; and

"WHEREAS, No precautions are exercised in this State to prevent the spreading of this disease; therefore, be it

"*Resolved,* That a committee of five or more be appointed to secure, if possible, such legislation as will result in the care and isolation of indigent consumptives in institutions built and maintained by the State."

Dr. Paschal's resolution was seconded and carried.

Dr. M. M. Smith offered the following resolution, which was seconded and carried:

"WHEREAS, The State of Texas has a medical department of the University of Texas located at Galveston, and under the control of the Board of Regents of the University of Texas.

"WHEREAS, This is the only time in the history of the institution that there has not been one or more physicians as members of the Board of Regents; and

"WHEREAS, The welfare of the medical and pharmacal departments of the State University requires the presence of one or more physicians on said board who are familiar with the needs and requirements of such institution; therefore, be it

"*Resolved,* That the Texas State Medical Association urge upon the Governor the importance of such representation in the Board of Regents, and the President of this Association is hereby instructed to bring the matter to the notice of the Governor, and urge the appointment of a physician on the Board of Regents of the University of Texas."

The Section on Gynecology was then declared open, with Dr. Fort in the chair and Dr. Rosser as secretary.

Dr. T. J. Bell, of Tyler, presented a paper entitled "How Much Have Obstetricians Contributed to the Work of the Gynecologist?"

The paper was, on motion, received and discussed.

A telegram was read from Dr. Henry Schwarz, of St. Louis, Mo., stating that owing to the sickness of his wife he was unable to attend the convention, but had sent his paper, entitled "Extra Uterine Pregnancy," to the Secretary.

Dr. J. E. Gilcreest, of Gainesville, presented a paper entitled "Report of a Case of Ectopic Gestation."

On motion, the paper was opened for discussion.

Dr. William Shropshire presented a paper entitled "Hypertrophic Prolongation of the Cervix," which was received and discussed.

A report of the Judicial Council was received and adopted, in which report Hon. William L. Prather, President of the University of Texas, was named as an honorary member of the Association.

Dr. West moved that all papers be referred to the Publishing Committee, and that the Chairman of all Sections write to the gentlemen who have had their names placed upon the program and urge them to send in their papers.

Motion carried.

The Section on Gynecology was then declared closed.

Dr. West moved that the convention go into executive session.

Motion prevailed.

The Chair then appointed the following campaign committee: J. M. Fort, chairman; C. M. Rosser, R. H. Harrison, W. Shropshire, J. W. Scott.

The Chair appointed the following committee under Dr. Paschal's resolution concerning Sanitaria for indigent consumptives: Dr. Frank Paschal, D. I. Fly, M. M. Smith, Taylor Hudson, and F. M. Pitts, Jr.

THE CHAIR: You will hear the report of Committee No. 2 on Public Health.

The following report was submitted:

"*To the President and Members of the Texas State Medical Association:*

"GENTLEMEN: Your committee charged with the duty of formulating and urging before the Legislature of the State a law providing for the formation of a Board of Health and providing for a record of the vital statistics of the State, has now completed its second year's service on this subject. The history of its efforts during its first year was submitted to the last session of the Association in San Antonio; and resulted in a memorial to His Excellency, the Governor, and members of the Legislature urgently soliciting their attention for this important subject. The arguments used in support of the measure were unanswerable and deemed entirely sufficient, but the executive ear was difficult to reach. The memorial met with no response from his excellency.

"When it became evident that an extraordinary session of the Legislature would be held, your committee had some hopes that the palpable necessity for a revision of our health laws would enable it to secure legislative action. Smallpox had been introduced into the State early in the preceding year, and had steadily spread its loathsome influence over the State despite the most vigorous efforts of the State Health Officer. The insufficiency of existing legislation was painfully manifest. There was no concert of action between the several counties of the State in its efforts to stay the spread of this foul pestilence, and it extended its baneful influence with very little hindrance; but the preoccupation of members of the Legislature with the financial question submitted to them barred the consideration of any other question, and your committee is again compelled to report failure.

"There seems to be a widespread and general misapprehension in the public mind as to the motives which impel the medical profession to interest itself in questions of this character. The unselfish desire of medical men to protect the public health, not only from imported pestilence, but from all such preventible diseases as has origin from local causes within the limits of the State is not appreciated. The duty of a government to protect the lives and promote the prosperity of its citizens is acknowledged; but only to the extent that it may be threatened from imported pestilence is it done.

"After it became evident that the elaborate and comprehensive law prepared by your committee and submitted to the Legislature would not be reported, another and much more simple draft, as well as less effective, was prepared and submitted to the Legislature as a substitute; but this occurred so late in the session that no action was obtained. Your committee now has some assurance that if that substitute meets with the approval of this Association it will secure the endorsement of the State Health Officer and the favor of his excellency the Governor. Your committee beg leave to submit a copy of that proposed law to the Association for its approval, reserving the right to make some slight alterations and additions before it goes to the Legislature, but which will not materially alter its scope or purpose. With the permission of the Association I will now read the proposed act. [Act omitted for want of space.—Ed.]

"All of which is respectfully submitted.

"M. M. Smith,
"Bacon Saunders,
"S. C. Red."

Dr. West moved the adoption of the report and the continuance of the committee.

The motion prevailed.

Dr. West moved that the last clause of Article X, of the by-laws be amended to read as follows: "The Treasurer shall receive for his services one hundred and fifty dollars."

Under the rules the proposed amendment was laid on the table until the next meeting.

The following report was presented and, on motion, received:

"*Dr. A. B. Gardner, President:*

"Your committee to whom was referred the paper read by Dr. West on the 'Maladministration of Public Medical Affairs in Texas,' and the address on 'State Medicine,' by M. M. Smith, together with the discussion of said papers, with instructions to cause the publication of same, beg leave to report that the time for adjournment being so near at hand, we find it inexpedient to submit a report to this body covering even in part the important subject, and therefore respectfully ask that we be given discretion in the matter of compiling, editing and printing said matter, pledging ourselves to give the subject our immediate and careful attention.

"R. H. HARRISON,
"F. E. DANIEL."

It was then moved that a nominating committee be appointed, the names to be selected by the different districts and handed to the Secretary to be announced as the first business of the afternoon session.

The motion prevailed.

The convention adjourned until 2:30 p. m.

### THIRD DAY.—*Afternoon Session.*

The nomination committee was appointed.

[Names omitted.]

The Section on Ophthalmology was then declared open.

Dr. W. R. Thompson, of Fort Worth, presented a paper entitled "Foreign Body in the Globe, with Extraordinary History," which was, on motion, received and discussed.

Dr. E. P. Daviss, of Houston, presented a paper entitled "Report of Cases Illustrating the Extremes in Special Surgery—Boldness and Conservatism."

The paper was, on motion, received and discussed.

Dr. Joseph Mullen, of Houston, read a paper entitled "The Use of Mercurol as a Valuable Non-Irritating Antiseptic in Intraocular Suppurative Processes," which was, on motion, received and opened for discussion.

Dr. R. F. Miller, of Sherman, read a paper entitled "The Present Status of Ear Work," which was, on motion, received and opened for discussion.

Dr. W. L. Rogers read a Report of Cases, which was, on motion, received and opened for discussion.

The Section on Ophthalmology was then declared closed.

THE CHAIR: The Constitution requires, and it is nothing but right, that we should devote at least one hour to a memorial service, which will now be called. Dr. Harrison is chairman of the Committee on Necrology. He will please come forward and preside.

Dr. Harrison took the chair.

[Memorial Services omitted.]

Dr. Paschal introduced the following resolution:

"WHEREAS, The Marine Hospital Service has taken the initiative in the matter of establishing special sanitaria in the Southwest for the care and treatment of indigent consumptives; and

"WHEREAS, This service, representing the general government, and with ample funds at its disposal, has promptly and cheerfully responded to all calls for aid in the matter of preventing the introduction and spread of contagious diseases in Texas and other States.

"*Resolved,* That this Association desires to hereby express its appreciation of the work of said Marine Hospital Service, and to aid and co-operate with them. We also invite the co-operation of the service in our efforts in the same cause, as per resolution upon the report of the West Texas Medical Society adopted this day.

"*Resolved,* That the Marine Hospital Service be and is hereby invited to send annually a representative to our State Medical Association.

"*Resolved,* That a copy of this resolution be sent to Supervising Surgeon-General Wyman."

DR. WEST: I rise to second these resolutions, and I do it with a great deal of pleasure. I think the resolutions are eminently in order and proper. We often forget the Marine Hospital Service until we get in serious trouble, and then we are very anxious to have their aid and co-operation, and as I say, I heartily endorse the resolutions and second them.

DR. KINGSLEY: I suggest we hear from Dr. Spohn, as a representative of the Marine Hospital Service.

[Dr. Spohn begun a speech but was called away before he had finished.—ED.]

Dr. Paschal's motion was then carried.

It was moved that the Section on Surgery be reopened, and the motion prevailed.

Dr. Kingsley, of San Antonio, presented his report as Chairman of the Section, entitled "A Brief Review of the Progress of Surgery for the Past Year."

Dr. Frank Paschal, of San Antonio, presented a paper entitled "Stone in the Bladder and Lithotomy."

[This paper will be published in our June number, with photographic illustration of fourteen of the stones.—ED.]

On motion, the paper was received and opened for discussion.

On motion, the convention adjourned until 8 p. m.

### THIRD DAY.—*Night Session.*

The President delivered his annual address.

Dr. F. E. Daniel delivered a eulogy on the life and services of the late Dr. Swearingen (published herewith), and Dr. J. D. Becton delivered the Annual Oration.

### FOURTH DAY.—*Morning Session.*

The nominating committee presented the following report:

"*Mr. President and Gentlemen of the State Medical Association:*

"We, your Nominating Committee, beg leave to submit the following report:

For President, Dr. B. E. Hadra, Waco; Dr. S. C. Red, Houston.

First Vice-President, Dr. Taylor Hudson, Belton.

Second Vice-President, Dr. L. Ashton, Dallas.

Third Vice-President, Dr. S. T. Turner, El Paso.

Secretary, Dr. H. A. West, Galveston.

Treasurer, Dr. R. F. Miller, Sherman.

Judicial Council, Dr. J. C. Anderson, Granger; Dr. T. J. Bell, Tyler; Dr. A. E. Spohn, Corpus Christi; Dr. W. A. Wood, Hubbard.

Orator, J. S. Price, Beaumont.

Chairman Committee of Arrangements, Henry P. Cooke, Galveston.

Place of meeting, Galveston.

Time of meeting, fourth Tuesday in April, 1901.

Dr. A. P. Daviss of Houston, Delegate to International Congress; Drs. R. F. Miller and F. O. Norris, alternates.

Respectfully submitted,

J. E. GILCREEST, Chairman.

J. S. PRICE, Secretary."

On motion, the report was received and the committee discharged.

A report of the Judicial Council was received and adopted.

The Chair appointed Drs. G. B. Foscue and M. M. Smith as tellers to count the vote for President, and, upon receiving and counting the vote, Dr. B. E. Hadra was declared elected President, and, on motion, his election was made unanimous.

It was moved that the remaining officers be declared elected unanimously as named in the report of the nominating committee.

The resolution prevailed unanimously.

On motion, the convention adjourned to meet at Galveston, Texas, on the fourth Tuesday in April, 1901.

